# Association between clinician-rated oral mucositis severity and oral mucositis-specific quality of life: A cross-sectional study

**DOI:** 10.1007/s00784-026-07278-9

**Published:** 2026-11-14

**Authors:** Pedro Henrique Gonçalves Ferreira, Lucas Guimarães Abreu, Gabriella Guerra Freire Gabrich, Natália Silva de Meira, Fabiana Maria Kakehasi, Tarcília Aparecida da Silva, Flávio de Freitas Mattos, Denise Vieira Travassos

**Affiliations:** 1 Professional Master of Public Health Dentistry, School of Dentistry, Federal University of Minas Gerais (UFMG), Belo Horizonte, Brazil; 2 Department of Child and Adolescent Oral Health, School of Dentistry, Federal University of Minas Gerais (UFMG), Belo Horizonte, Brazil; 3 Multiprofessional Integrated Residency in Health, Hospital das Clínicas, Federal University of Minas Gerais (UFMG), Belo Horizonte, Brazil; 4 Department of Pediatrics, School of Medicine, Federal University of Minas Gerais (UFMG), Belo Horizonte, Brazil; 5 Department of Oral Surgery, Pathology and Clinical Dentistry, School of Dentistry, Federal University of Minas Gerais (UFMG), Belo Horizonte, Brazil; 6 Department of Social and Preventive Dentistry, School of Dentistry, Federal University of Minas Gerais (UFMG), Av. Presidente Antônio Carlos, 6627, Belo Horizonte, MG 30270-901 Brazil

**Keywords:** Stomatitis, Quality of life, Patient reported outcome measures, Oral health, Antineoplastic protocols, Symptom assessment

## Abstract

**Purpose:**

To evaluate the association between clinician-rated oral mucositis (OM) severity and oral mucositis–specific quality of life (OMQoL) in adults undergoing antineoplastic therapy or hematopoietic stem cell transplantation.

**Methods:**

This cross-sectional study included 56 adults undergoing chemotherapy, hematopoietic stem cell transplantation or head and neck radiotherapy with or without concurrent systemic chemotherapy at a Brazilian public university hospital. OM was assessed clinically according to the World Health Organization (WHO) grading scale, and oral mucositis specific quality of life (OMQoL) was assessed using the shortened Brazilian version of the questionnaire, comprising four domains: Diet, Symptoms, Social Functioning, and Swallowing. Associations between WHO grades and OMQoL scores were analyzed using non-parametric tests. Descriptive and bivariate analyses (Mann-Whitney and Kruskal-Wallis tests) was performed to evaluate associations between clinical variables and QoL scores (*p* < 0.05).

**Results:**

Among the 56 participants, 32 (57.1%) were female, and the mean age was 51 years. WHO grades 3 and 4 were observed in 29 participants (51.8%). OMQoL scores differed significantly according to WHO grade across all domains (*p* = 0.001), with mean total scores ranging from 24.36 among participants with grade 0 OM to 48.67 among those with grade 4 OM. Female participants had higher OMQoL scores in the Diet domain (*p* = 0.023), although this finding should be interpreted as exploratory. Age and diagnostic category were not significantly associated with OMQoL scores. The sensitivity analysis excluding patients with non-malignant hematologic disease yielded similar findings.

**Conclusion:**

Higher clinician-rated WHO grades of OM were associated with poorer OM-specific quality of life in this sample. The association was particularly relevant for domains related to diet, symptoms, social functioning, and swallowing. Because WHO grading incorporates functional criteria related to oral intake, the observed associations should be interpreted considering the partial overlap between clinician-rated OM severity and patient-reported functional impairment. Longitudinal studies are warranted to clarify temporal relationships between OM severity and quality of life.

**Supplementary Information:**

The online version contains supplementary material available at https://doi.org/10.1007/s00784-026-07278-9.

## Introduction

Oral mucositis (OM) is one of the most prevalent and debilitating complications of antineoplastic treatment, particularly among patients undergoing chemotherapy, head and neck radiotherapy, and hematopoietic stem cell transplantation [1–5]. The condition is characterized by inflammation and ulceration of the oral mucosa, resulting in severe pain and impairment of speech and swallowing functions. In addition to increasing the risks of infection and malnutrition, such complications may lead to treatment interruption and compromised clinical prognosis [6–9].

Beyond local manifestations, oral mucositis has been associated with systemic and psychosocial consequences, including impaired nutritional status, treatment adherence, and deterioration in health-related quality of life [6, 10–12]. Pain and oral inflammation have been associated with poorer quality of life, particularly through their interference with basic functions such as eating, swallowing, and communication [13–18]. Consequently, the assessment of mucositis should extend beyond the clinical severity of the lesions to include subjective aspects of patient-reported symptoms, considering the physical, functional, and emotional dimensions affected by treatment [19, 20].

The high complexity of antineoplastic therapies and the significant healthcare burden require integrated multidisciplinary strategies. In this setting, the dentist plays an important role in the prevention, early diagnosis, and management of oral toxicities resulting from treatment. Dental care may contribute to symptom management, preservation of oral function, and supportive care throughout the therapy [21, 22]. Thus, integrating clinical assessment with patient-reported outcomes can provide a broader understanding of mucositis and its association with quality of life.

Given this context, the present study aimed to evaluate the association between clinician-rated oral mucositis severity and oral mucositis-specific quality of life among patients undergoing antineoplastic therapy or stem cell transplantation at a public university hospital in Brazil, within the Unified Health System (SUS). Specifically, the study sought to characterize the association between the clinician-rated oral mucositis and patient-reported symptoms and functional and psychosocial aspects of oral mucositis-specific quality of life.

## Methods

### Study design and participants

This is an observational, cross-sectional, and quantitative study conducted at the Hospital das Clínicas of the Universidade Federal de Minas Gerais (HC-UFMG), a major oncology referral center in Belo Horizonte, Brazil. Data collection took place between March 2024 and April 2025.

A convenience sampling strategy was used. Patients referred to the hospital dentistry service during the study period, including hospitalized patients and outpatient clinic attendees, were screened for eligibility. Inclusion criteria were: (1) age ≥ 18 years; and (2) undergoing cytotoxic chemotherapy, head and neck radiotherapy with or without concurrent systemic therapy, or conditioning for hematopoietic stem cell transplantation (HSCT). Patients with non-malignant conditions undergoing treatment associated with a risk of oral mucositis were also eligible; therefore, eligibility was based on treatment exposure rather than exclusively on the presence of a malignant diagnosis. Exclusion criteria were cognitive or communication impairments that prevented completion of the study procedures and refusal to participate.

For analysis, participants were classified into three diagnostic categories: hematological malignancies, solid malignancies, and non-malignant conditions. A sensitivity analysis excluding participants with non-malignant conditions was performed to assess whether their inclusion materially affected the findings (Supplementary Table S3).

Participants were clinically followed during the period of risk for oral mucositis. When oral mucositis was identified, WHO grading and OMQoL assessment were performed at the same assessment and repeated if the WHO grade subsequently changed. For participants with more than one paired assessment, only the WHO–OMQoL assessment corresponding to the highest WHO grade observed during follow-up was retained for analysis; earlier paired assessments were excluded. Thus, the analytical dataset contained a single paired WHO–OMQoL observation per participant.

Oral health conditions were recorded at first dental examination; dental treatment were performed only in cases where the systemic patient conditions were favorable. However, in many cases these patients were referred to dental service after chemotherapy and/or radiotherapy had already been initiated due to healthcare system workflows or the severity of the condition.

### Instrument

Oral mucositis–related quality of life was assessed using the shortened version of the Oral Mucositis Quality of Life (OMQoL) questionnaire [15–17], specifically adapted and validated for the Brazilian context [19, 20] (Supplementary File 1). This instrument comprises 17 items across four domains: Diet, Symptoms, Social Functioning, and Swallowing. Higher scores reflect greater impairment of quality of life. The original version developed by Cheng et al. [15–17] is also available as supplementary material (Supplementary File 2).

### Data collection and procedures

Data collection involved direct clinical assessments and face-to-face individual interviews conducted in person by a single researcher, taking approximately 25 min per participant. Participants received standardized instructions, and clarifications were provided without influencing responses. No OMQoL items were missing.

Participants were followed clinically throughout the risk period for oral mucositis until complete lesion resolution. During each evaluation, the WHO clinical grade was determined first, followed immediately by administration of the OMQoL questionnaire. For participants who did not develop clinically detectable oral mucositis during follow-up, the assessment was performed between 12 and 15 days post-treatment, corresponding to the peak risk period for lesion appearance [1, 2].

For this cross-sectional analysis at the individual level, only a single paired WHO–OMQoL observation per participant was analyzed, corresponding to the highest WHO grade recorded during follow-up.

### Clinical assessment and study variables

Clinical assessment of oral mucositis was performed by a single researcher according to the World Health Organization (WHO) grading scale: Grade 0 – No changes (Normal); Grade 1 – Erythema and mild pain; Grade 2 – Erythema and ulceration, but able to eat solids; Grade 3 – Extensive ulceration requiring a liquid diet only; and Grade 4 – Oral intake is not possible [23, 24]. Because the WHO scale incorporates both clinical manifestations and functional criteria related to oral intake, its functional components partially overlap with the Diet and Swallowing domains of the OMQoL. This conceptual overlap was considered when interpreting the association between WHO mucositis severity and OMQoL scores.

In addition to the mucositis grading, were collected using a structured form and through medical record reviews, sociodemographic and clinical data (age, sex, education level, marital status, primary diagnosis, disease status, treatment modality and regimen, and, when applicable, chemotherapy cycle and timing relative to the retained assessment; HSCT type, conditioning regimen and intensity, total-body irradiation, and day relative to transplantation; and radiotherapy site, prescribed dose, fractionation, cumulative dose at assessment, and concurrent systemic therapy and treatment-related characteristics). Additional clinical variables available for the retained assessment included OM onset and duration, baseline oral health, number and anatomical location of oral lesions, presence of oral candidiasis or other infections, odynophagia, xerostomia, dysgeusia, feeding-tube use, and number of photobiomodulation therapy (PBMT) sessions. All participants received prophylactic and/or therapeutic PBMT according to institutional protocols.

Oral health conditions at the initial dental examination, including dental/periodontal findings and treatment indications, are detailed in Supplementary Table S1.

### Statistical analysis

Data were tabulated using Microsoft Excel^®^ and analyzed using the Statistical Package for the Social Sciences (SPSS^®^) software, version 22.0 (IBM Corp., Armonk, NY, USA). Descriptive analysis was performed, with absolute and relative frequencies presented for categorical variables, while quantitative variables were expressed as means, standard deviations, and medians.

Due to the non-normal distribution of data, non-parametric tests were employed to assess associations between sociodemographic and clinical variables and the OMQoL domain scores. The Mann-Whitney test was used for comparisons between two groups, and the Kruskal-Wallis test was used for comparisons involving more than two groups. We also conducted these analyses after removing individuals with non-malignant conditions. The level of statistical significance was set at 5% (*p* < 0.05). Effect sizes (ES) along with their 95% confidence intervals (CI) were also calculated. ES around 0.20 were considered small. Those around 0.60 were moderate and ES around 0.80 or higher were large [25].

### Ethical considerations

This study was conducted in accordance with the ethical principles for research involving human subjects, following Resolution N^o^. 466/2012 of the Brazilian National Health Council (CNS). The protocol was approved by the Research Ethics Committee of the Federal University of Minas Gerais (CEP-UFMG), under CAAE number 75034923.0.0000.5149, with institutional approval from HC-UFMG/EBSERH (Letter SEI nº. 144/2023/SGPITS/GEP/HC-UFMG-EBSERH).

All participants were fully informed about the objectives, procedures, risks, and benefits of the study. Their right to withdraw from research at any time without prejudice to their clinical treatment was guaranteed. Informed consent (ICF) was obtained in writing from all participants. The authors declare no conflict of interest regarding this study.

## Results

### Sociodemographic and clinical characteristics of the sample

During the study period, 61 patients were referred to for dental evaluation. Although all met the eligibility criteria given the characteristics of the hospital service, five refused to participate, resulting in a final sample of 56 patients, comprising inpatients (*n* = 47, 83.8%) and outpatients (*n* = 9, 16.2%) under follow-up at the hospital dental service. Table 1 summarizes the sociodemographic and clinical profile of the sample, including sex, age group, education level, primary diagnosis, therapeutic protocol, and oral mucositis grade.

A female predominance was observed (57.1%), with a mean age of approximately 51 years (range: 20–76 years). The 40–59 age group was the most representative (39.3%). Regarding education, most participants completed high school (42.9%).

Participants were categorized into: (1) malignant hematologic diseases (*n* = 37, 66.1%), (2) solid malignancies (*n* = 14, 25.0%), and (3) non-malignant conditions or neoplasms of uncertain behavior (*n* = 5, 8.9%). Patients with sickle cell disease were included because they received stem cell transplantation conditioning regimens, and one patient with a peripheral nerve neoplasm of uncertain behavior was included for undergoing a conventional chemotherapy regimen, both treatment modalities carrying a potential risk of mucositis development.

Regarding treatment, the participants were classified into: (1) systemic chemotherapy (*n* = 26, 46.4%) (2) HSCT conditioning (*n* = 24, 42.9%) (3) Concurrent chemoradiotherapy (*n* = 5, 8.9%) and (4) radiotherapy (*n* = 1, 1.8%).

Regarding oral mucositis, most participants presented some degree of lesion. Grades 3 and 4, considered severe oral mucositis, were the most prevalent, accounting for 51.8% of cases. Grade 2 was observed in 17.9%, while Grades 1 and 0 accounted for 10.7% and 19.6%, respectively.

**Table 1:** Sociodemographic and clinical characteristics of the patients included in the study, Hospital das Clínicas of the UFMG, Belo Horizonte/MG, March/2024 to April/2025 (n=56)

Variables	n (%)
Sex	
Female	32 (57.1)
Male	24 (42.9)
Age	
< 40	20 (35.7)
40-59	22 (39.3)
≥ 60	14 (25.0)
Educational Level	
Incomplete Primary Education	11 (19.6)
Complete Primary Education	09 (16.1)
Incomplete Secondary Education	03 (05.4)
Complete Secondary Education	24 (42.9)
Incomplete Higher Education	01 (01.8)
Complete Higher Education	06 (10.7)
Complete Postgraduate Education	02 (03.6)
Diagnosis	
malignant hematologic diseases	37 (66.1)
solid malignancies	14 (25.0)
non-malignant conditions	05 (8.9)
Treatment	
Systemic Chemotherapy	26 (46.4)
HSCT conditioning	24 (42.9)
Concurrent chemoradiotherapy	05 (8.9)
Radiotherapy	01 (1.8)
WHO oral mucositis grade	
0	11 (19.6)
1	06 (10.7)
2	10 (17.9)
3	23 (41.1)
4	06 (10.7)
*Severe oral Mucositis (WHO grades 3-4)	29 (51.8)

### Treatment characteristics of the study participants

Table 2 summarizes the treatment characteristics of the study participants (* n=56*). Regarding treatment modalities, 26 participants (46.4%) received systemic antineoplastic therapy, 24 (42.9%) underwent HSCT conditioning, 5 (8.9%) received concurrent head and neck chemoradiotherapy, and 1 (1.8%) received head and neck radiotherapy alone. Among those who underwent HSCT conditioning (* n=24*), the majority received autologous HSCT (* n=17*, 70.8%) and myeloablative conditioning (* n=20*, 83.3%). The most frequent conditioning regimens were Melphalan-based without other agents (* n=10*, 41.7%), Busulfan + Fludarabine + ATG (* n=7*, 29.2%), and Busulfan + Melphalan (* n=5*, 20.8%). Total-body irradiation (TBI) was administered to 1 participant (4.2%).

For participants receiving systemic antineoplastic therapy (* n=26*), the median interval from the last systemic treatment to the retained assessment was 10.5 days (IQR: 5.25–13.5). Among participants receiving head and neck radiotherapy (* n=6*), 5 (83.3%) received concurrent systemic therapy. A prescribed schedule of 35 fractions was used in 5 patients (83.3%), and half of the radiotherapy group (* n=3*, 50.0%) reached a cumulative dose of 70 Gy at the retained assessment. Additional lesion-oriented clinical findings and mucositis-related symptoms recorded at the retained assessment are summarized in Supplementary Table S2.

**Table 2:** Treatment characteristics of the study participants (n = 56)

Treatment characteristic	n (%)
Treatment modality	
Systemic antineoplastic therapy	26 (46.4)
HSCT conditioning	24 (42.9)
Concurrent head and neck chemoradiotherapy	5 (8.9)
Head and neck radiotherapy alone	1 (1.8)
HSCT characteristics (n = 24)	
Autologous HSCT	17 (70.8)
Allogeneic HSCT	7 (29.2)
Myeloablative conditioning	20 (83.3)
Reduced-intensity conditioning	4 (16.7)
TBI	1 (4.2)
Conditioning regimen (n = 24)	
Melphalan-based, without other conditioning agents	10 (41.7)
Busulfan + fludarabine ± ATG	7 (29.2)
Busulfan + melphalan	5 (20.8)
BEAM	1 (4.2)
Cyclophosphamide + busulfan	1 (4.2)
Systemic antineoplastic therapy (n = 26)	
Treatment regimen/phase documented	26 (100.0)
Days from last systemic treatment to retained assessmentMedian (IQR)	10.5(5.25–13.5)
Range, days	1–75
Head and neck radiotherapy (n = 6)	
Concurrent systemic therapy	5 (83.3)
Radiotherapy alone	1 (16.7)
35 prescribed fractions	5 (83.3)
29 prescribed fractions	1 (16.7)
Cumulative RT dose at retained assessment (n = 6)	
20 Gy	1 (16.7)
24 Gy	1 (16.7)
33.75 Gy	1 (16.7)
70 Gy	3 (50.0)

Percentages within HSCT and radiotherapy sections use their respective subgroup denominators. *ATG* antithymocyte globulin; BEAM, carmustine, etoposide, cytarabine, and melphalan, *HSCT* hematopoietic stem cell transplantation, *IQR* interquartile range, *RT* radiotherapy, *TBI* total-body irradiation

### Association between clinical characteristics and quality of life

Table 3 shows the analysis of associations between clinical and sociodemographic variables and the scores obtained in the Brazilian version of the Oral Mucositis Quality of Life (OMQoL) instrument.

In an unadjusted analysis, female patients had higher mean scores in the Symptoms and Diet domains, as well as in the total score, compared to males. A statistically significant difference was observed in the ‘Diet’ domain (* p=0.023*), indicating a greater association of oral mucositis on dietary aspects among women. The ES for this domain was 0.61 (moderate). This finding should be interpreted as exploratory, as it may be influenced by multiple factors not controlled for in this specific analysis.

No significant differences were found across age groups, suggesting that age was not a determining factor in the perception of oral mucositis-related quality of life. Similarly, no significant association was observed between the type of diagnosis (hematologic vs. non-hematologic) and quality of life scores, although patients with non-hematologic diseases showed slightly higher means in the Swallowing and Social Functioning domains. The ES were small.

Regarding the therapeutic protocol, patients undergoing combined radiochemotherapy presented higher scores across all domains and in the total OMQoL score compared to those receiving chemotherapy alone. However, these differences did not reach statistical significance (* p>0.05*).

**Fig. 1:**
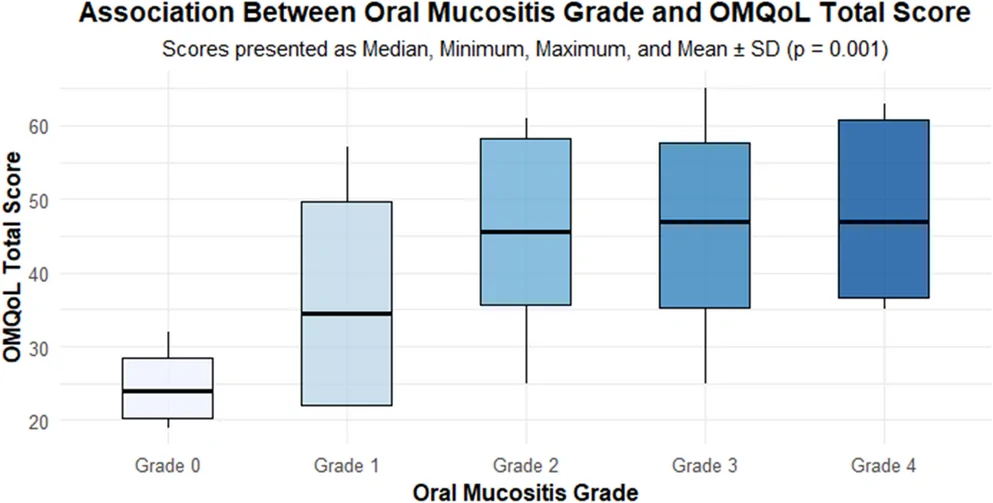
Association between Oral Mucositis WHO severity grades and OMQoL total score ($\:\boldsymbol{n}=56$). Note: Data are presented as medians (solid lines), minimum and maximum values (whiskers), and mean $\:\pm\:$ SD (box boundaries). A statistically significant difference in quality of life scores was observed across oral mucositis severity grades (* p=0.001*, Kruskal-Wallis test)

**Fig. 2:**
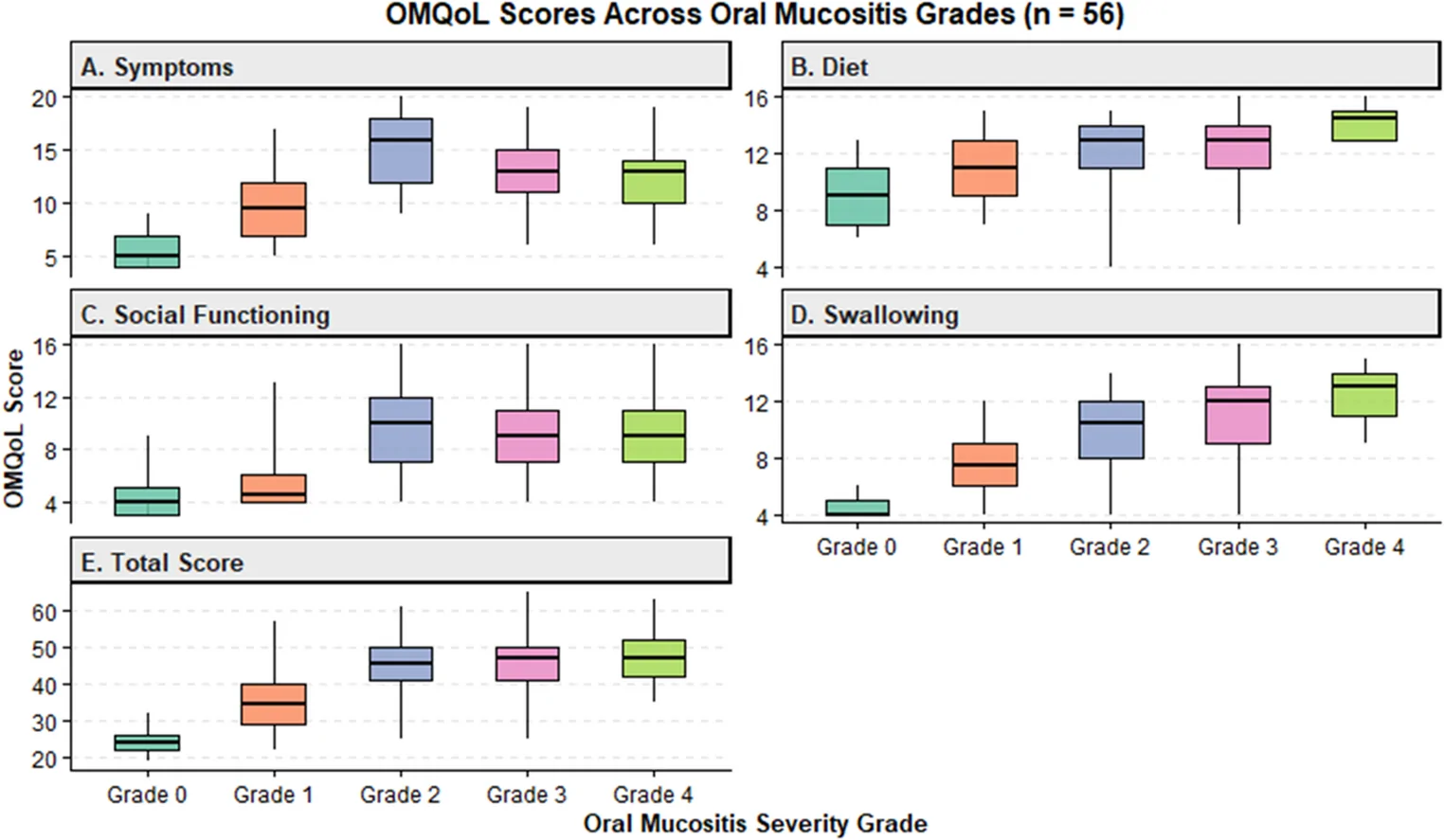
Oral Mucositis Quality of Life (OMQoL) domain and total scores according to Oral Mucositis WHO severity grades ($\:\mathbf{n}=56$). Note: Scores are expressed as medians, minimums, and maximums. Significant differences across grades were observed for all domains and total score ($\:\mathrm{p}<0.05$, Kruskal-Wallis test)

The oral mucositis grade showed statistically significant differences across all evaluated domains (𝑝 = 0.001), with higher OMQoL scores generally observed among participants with more severe mucositis. Mean total scores increased from 24.36 in Grade 0 to 48.67 in Grade 4 (Fig. 1), indicating that patients with grades 3 and 4 mucositis experienced the greatest quality of life impairment. Furthermore, higher WHO severity grades were associated with poorer outcomes across all individual OMQoL domains (Diet, Symptoms, Social Functioning, and Swallowing) as well as the overall total score (Fig. 2). Effect sizes ranged from 0.65 to 4.15, with most ES being well above 0.80 (large to very large).

Supplementary File 1 displays the results of the analyses after removing the individuals with non-malignant conditions from the dataset. The results were similar to those when the total sample is analyzed.​

A se​nsitivity analysis excluding participants with non-malignant conditions yielded consistent results with the main analysis (Supplementary Table S3).

**Table 3:** Association between sociodemographic and clinical variables and OMQoL domain and total scores, Hospital das Clínicas of the UFMG, Belo Horizonte/MG, March/2024 to April/2025 (n=56)

	**Symptoms** Mean (SD) Median(Min-Max)	ES(95% CI)	*p*	**Diet** Mean (SD) Median(Min-Max)	ES(95% CI)	*p*	**Social Functioning ** Mean (SD) Median(Min-Max)	ES(95% CI)	*p*	**Swallowing** Mean (SD) Median(Min-Max)	ES(95% CI)	*p*	**Total Score** Mean (SD) Median(Min-Max)	ES(95% CI)	*p*
Sex															
Female	12.78 (4.96) 13.5 (5-20)	0.44(0.18-0.70)	0.138	13.06 (2.85) 13.5 (7-16)	0.61(0.35-0.87)	0.023	8.34 (4.23) 7.5 (4-16)	0.08(-0.08-0.34)	0.754	9.22 (3.76) 8.5 (4-16)	0.11(-0.15-0.37)	0.713	43.41 (13.62) 45.0 (20-65)	0.36(0.10-0.62)	0.167
Male	10.54 (5.03) 10.0 (5-20)			11.17 (3.25) 11.5 (4-16)			8.00 (4.05) 6.0 (4-15)			8.79 (3.91) 8.5 (4-15)			38.50 (13.64) 35.5 (19-63)		
Age															
< 40	11.40 (5.21) 13.0 (5-19)		0.705	12.10 (3.08) 12.5 (6-16)		0.916	8.45 (4.10) 9.0 (4-16)		0.941	9.45 (3.91) 9.5 (4-15)		0.801	41.40 (14.27) 40.5 (19-62)		0.978
40-59	12.55 (5.12) 13.0 (5-20)	0.22(-0.04-0.48)		12.32 (3.52) 13.5 (4-16)	0.06(-0.20-0.32)		7.82 (4.19) 6.0 (4-16)	0.15(-0.11-0.41)		9.00 (4.22) 8.5 (4-16)	0.11(-0.15-0.37)		41.68 (14.10) 42.0 (20-65)	0.02(-0.24-0.28)	
≥ 60	11.29 (5.05) 11.0 (5-19)	0.02(-0.24-0.28)		12.36 (2.82) 13.0 (7-16)	0.08(-0.18-0.34)		8.43 (4.31) 8.5 (4-16)	0.01(-0.25-0.27)		8.50 (3.06) 7.0 (5-15)	0.27(0.01-0.53)		40.57 (13.34) 40.5 (23-63)	0.06(-0.20-0.32)	
Diagnosis															
Hematologic	11.89 (5.22) 13.0 (5-20)	0.06(-0.20-0.32)	0.882	12.11 (3.15) 13.0 (4-16)	0.22(-0.04-0.48)	0.456	7.82 (4.09) 6.0 (4.16)	0.46(0.20-0.72)	0.170	8.98 (3.82) 9.0 (4-16)	0.07(-0.19-0.33)	0.716	40.80 (14.04) 40.0 (19-65)	0.19(-0.07-0.45)	0.577
Nonhematologic	11.56 (4.63) 13.0 (5-19)			12.82 (3.22) 13.0 (7-16)			9.73 (4.08) 10.0 (4-16)			9.27 (3.90) 8.0 (4-15)			43.36 (12.79) 44.0 (25-63)		
Cancer Treatment															
Chemotherapy	11.72 (4.94) 13.0 (5-20)	0.14(-0.12-0.40)	0.617	12.10 (3.25) 13.0 (4-16)	0.55(0.29-0.81)	0.423	8.02 (4.07) 6.5 (4-16)	0.37(0.11-0.63)	0.473	8.82 (3.77) 8.0 (4-16)	0.52(0.26-0.78)	0.185	40.66 (13.70) 40.50 (19-65)	0.43(0.17-0.69)	0.352
Concurrent chemoradiotherapy	12.57 (6.56) 13.0 (5-20)			13.50 (1.76) 13.0 (11-16)			9.67 (4.68) 11.0 (4-15)			10.83 (3.87) 11.5 (5-15)			46.67 (13.94) 44.5 (32-63)		
	**Symptoms** Mean (SD) Median (Min-Max)	ES(95% CI)	*p*	**Di** **et** Mean (SD) Median (Min-Max)	ES(95% CI)	*p*	**Social Functioning** ​Mean (SD) Median (Min-Max)	ES(95% CI)	*p*	**Swallowing** Mean (SD) Median (Min-Max)	ES(95% CI)	*p*	**Total Score** Mean (SD) Median (Min-Max)	ES(95% CI)	*p*
Oral Mucositis															
Grade 0	5.82 (1.32) 5.0 (5-9)^a^		0.001	9.36 (2.54) 9.0 (6-13)^a^		0.003	4.45 (1.50) 4.0 (4-9)^a^		0.001	4.73 (0.90) 4.0 (4-6)^a^		0.001	24.36 (4.08) 24.0 (19-32)^a^		0.001
Grade 1	10.33 (5.92) 9.5 (5-17)^ab^	1.24(0.98-1.50)		11.33 (3.44) 11.0 (7-15)^ab^	0.65(0.39-0.91)		6.33 (3.61) 4.5 (4-13)^ab^	0.73(0.47-0.99)		7.83 (2.63) 7.5 (4-12)^ab^	1.75(1.49-2.01)		35.83 (13.84) 34.5 (22-57)^ab^	1.28(1.02-1.54)	
Grade 2	15.50 (3.34) 16.0 (9-20)^b^	4.15(3.89-4.41)		12.40 (3.43) 13.0 (4-15)^ab^	1.02(0.76-1.38)		9.50 (4.57) 10.0 (4-16)^b^	1.66(1.40-1.92)		9.50 (3.62) 10.5 (4-14)^b^	2.11(1.85-2.37)		46.90 (11.29) 45.5 (25-61)^b^	2.93(2.67-3.19)	
Grade 3	13.39 (4.05) 13.0 (6-19)^b^	2.82(2.56-3.08)		13.17 (2.65) 13.0 (7-16)^b^	1.47(1.21-1.73)		9.57 (3.67) 9.0 (4-16)^b^	1.98(1.72-2.24)		10.35 (3.60) 12.0 (4-16)^b^	2.48(2.22-2.74)		46.48 (11.24) 47.0 (25-65)^b^	2.88(2.62-3.14)	
Grade 4	12.17 (5.60) 13.0 (6-19)^ab^	1.83(1.57-2.09)		14.67 (1.21) 14.5 (13-16)^b^	2.83(2.57-3.09)		9.50 (4.80) 9.0 (4-16)^b^	1.60(1.34-1.86)		12.33 (2.33) 13.0 (9-15)^b^	4.72(4.46-4.98)		48.67 (12.01) 47.0 (35-63)^b^	3.02(2.76-3.28)	

*S​D* standard deviation, *Min* minimum, *Max* maximum, *ES* effect size, *CI* confidence interval

Different letters (^a, ab, b^) indicate statistically significant differences according to the Kruskal-Wallis test for more than two categories of the independent variable

## Discussion

The results demonstrate that oral mucositis was highly prevalent among the evaluated oncology patients, exerting an association on quality of life, especially in domains related to eating and swallowing. Although variables such as age, sex, and type of malignancy did not show a significant association with OMQoL scores, the severity of oral mucositis was strongly associated with quality-of-life impairment (* p=0.001*; effect sizes ranged from * 0.65* to * 4.15*). These findings support the relevance of considering in addition to clinician evaluation, patient-report outcomes and reinforce the importance of preventive strategies and early management of this condition within the context of oncological treatment.

The present study found that greater oral mucositis severity was associated with greater impairment in oral mucositis-specific quality of life among adults undergoing antineoplastic treatment at HC-UFMG. Participants with moderate to severe OM (grades 2, 3, and 4) showed higher OMQoL scores across the Symptoms, Diet, Social Functioning, and Swallowing domains, as well as in the total score.

In this context, it is fundamental to differentiate the broad concept of “quality of life” from that applied in clinical practice, which is frequently restricted to “health-related quality of life” (HRQoL). HRQoL tends to focus on the mitigation of biological dysfunctions, using biostatistical and psychometric indicators to quantify the patient’s condition [26]. Although useful, this functionalist approach must be complemented by the WHO definition [27], which conceives QoL as the individual’s subjective perception within their cultural context and value system, recognizing that OM affects dimensions that transcend the physical aspect.

The perception of HRQoL is profoundly influenced by the social determinants of health. In a scenario of high social inequality, such as in Brazil, factors like income and education level shape the experience of the disease. The suffering resulting from OM can be intensified by pre-existing social vulnerabilities, which reinforces the need for interdisciplinary approaches that integrate clinical, social, and subjective aspects into the care of cancer patients.

A methodological consideration of this study is the inherent overlap between the WHO grading scale and the OMQoL instrument. Since WHO grades 2 to 4 are partially defined by functional dietary limitations, the OMQoL includes diet and swallowing domains that assess difficulties closely related to these functions. Therefore, the association found may reflect the complementary nature of clinician-rated and patient-reported assessments.

In our sample, WHO grade 0 patients, the mean OMQoL score was 24.36 (median: 24.0; range: 19–32). This value lies close to the theoretical scale floor of 17 (17 items $\:\times\:$ minimum score 1), indicating minimal impairment (mean item response of ~ 1.4). The slight deviation above the minimum score highlights the instrument’s sensitivity in capturing mild, non-ulcerative toxicities (e.g., sticky saliva, dysgeusia) not encompassed by the WHO visual scale.

The findings of this study corroborate national and international literature, which positions OM not only as a local toxicity but as a condition with systemic and psychosocial repercussions [28, 29]. The impairment in the ‘Diet’ and ‘Swallowing’ domains directly reflects the findings of the instrument’s developers [16, 17] and other studies in Brazil [19, 20, 30]. Severe functional impact often culminates in serious clinical outcomes, such as weight loss and the need for enteral nutritional therapy [31], and is associated with high levels of psychological distress, such as anxiety and depression [12, 32]. The centrality of pain, observed in studies using other instruments such as the OHIP-14 [10, 33], is captured by the OMQoL and possesses a measurable biological substrate, correlating with inflammatory biomarkers [34].

The use of OMQoL, a disease-specific instrument, proved pivotal. Literature indicates that generic QoL instruments may not adequately capture the specific clinical aspects of OM. This is illustrated by a study conducted in the same city (Belo Horizonte-MG, Brazil) [35], which, when using the EORTC QLQ-C30 and QLQ-H&N35, found no significant differences in global QoL despite the presence of severe OM. In contrast, the present study, by applying the shortened version of the OMQoL [19, 20], demonstrated sensitivity in measuring the effects of OM, proving to be a viable tool that is easy to apply and understand for clinical practice in high-demand services, such as the Unified Health System (SUS).

The study population was heterogeneous, patients were exposed to different treatments that differ substantially in their mechanisms of mucosal injury, toxicity and intensity. Eligibility was based on exposure to treatment associated with OM risk rather than exclusively on the presence of a malignant diagnosis.

A relevant aspect of the context of this study is that all participants received photobiomodulation therapy (PBMT) according to institutional protocols [36]. Because PBMT was provided to all participants, there was no untreated comparison group in the present study. Therefore, the study cannot determine whether PBMT influenced oral mucositis severity or OMQoL outcomes. Although previous studies at this institution [36, 37] and recent systematic reviews [38, 39] have reported benefits of PBMT for the prevention or management of oral mucositis, these findings cannot be directly inferred from the present study. The use of PBMT should therefore be considered when interpreting the observed associations between clinician-rated oral mucositis severity and patient-reported quality of life.

This study was conducted exclusively with adults, reflecting most investigations in the field. However, literature points to a significant gap in understanding the impacts of OM in pediatric patients [40, 41]. Future directions should include longitudinal studies to monitor the evolution of symptoms and the cumulative association to OM on QoL. Multicenter investigations are necessary to broaden the external validity of the findings, while qualitative approaches can deepen the understanding of the patient’s subjective experience, contributing to more humanized care.

This study presents limitations that must be considered. The sample size (*n* = 56) may have reduced the statistical power to identify associations in subgroups, and the cross-sectional design prevents the analysis of causality or the evolution of symptoms over time. Another critical limitation is the lack of examiner blinding; since the same researcher performed the clinical WHO grading and administered the OMQoL questionnaire, there is a potential risk of observer bias. Furthermore, as a single-center study (HC-UFMG), the results should be extrapolated with caution. The shortened version of the OMQoL, due to its recent incorporation, also lacks further exploration in the literature for comparative purposes.

Despite the limitations, this research offers relevant contributions to supportive care in oncology within the context of the SUS, suggesting an association between OM severity and QoL impairment, using a specific and adapted instrument, the study reinforces the need for rigorous preventive and therapeutic measures. The findings underline the critical need to integrate proactive dental management as an essential component of cancer patient care, aiming to mitigate physical and psychosocial suffering and qualify oncological assistance.

## Conclusion

In this cross-sectional study, clinician-rated WHO oral mucositis severity was associated with poorer patient-reported oral mucositis-specific quality of life across the OMQoL domains. The Diet and Swallowing domains warrant particular caution in interpretation because the WHO scale incorporates functional criteria related to oral intake, resulting in partial conceptual overlap between clinician-rated mucositis severity and patient-reported functional impairment. Accordingly, these measures should be considered complementary rather than interchangeable assessments of oral mucositis burden during antineoplastic therapy.

These findings underscore the clinical relevance of incorporating routine patient-reported outcome measures alongside clinician-rated assessments. Specialized hospital dentistry within multidisciplinary oncology teams may contribute to comprehensive supportive care, symptom management, and patient well-being during antineoplastic treatment, including in public health settings such as the SUS. Given the cross-sectional design and the heterogeneity of the study population, the observed associations should not be interpreted as evidence of causality.

## Supplementary Information

Below is the link to the electronic supplementary material.

Supplementary Material 1

## Data Availability

No datasets were generated or analysed during the current study.
