# Supplementary material for: Association between clinician-rated oral mucositis severity and oral mucositis-specific quality of life: A cross-sectional study
Source: Clin Oral Investig. 2026 Nov 14;30(12):577. doi: 10.1007/s00784-026-07278-9 (PMC13769721; doi:10.1007/s00784-026-07278-9)
Supplement: Supplementary file 1 [file 784_2026_7278_MOESM1_ESM.docx]

**SUPPLEMENTARY MATERIAL**

**Supplementary File 1. Brazilian shortened version of the Oral Mucositis Quality of Life (OMQoL) questionnaire - Portuguese items with English version.**

| **DIMENSÕES**  **Dimensions** | **CLASSIFICAÇÃO****  **Scores** | | | | **PONTUAÇÃO**  **Total score** |
| --- | --- | --- | --- | --- | --- |
|  | **1** | **2** | **3** | **4** |  |
| 1. **SINTOMAS**   **Symptoms** | | | | | |
| **(1) Tenho inchaço dentro da minha boca**  I have swelling inside my mouth |  |  |  |  |  |
| **(2) Tenho feridas / úlceras na boca**  I have mouth ulcer |  |  |  |  |  |
| **(3) Dores na boca me deixam angustiado**  Mouth pain makes me distressed |  |  |  |  |  |
| **(4) Tenho secreção / sangramento em meus lábios, ou dentro da minha boca**  I have oozing/bleeding on my lips, or inside my mouth |  |  |  |  |  |
| **(7) Tenho dores na boca**  I have mouth pain |  |  |  |  |  |
| 2. **DIETA**   **Diet** | | | | | |
| **(10) Não consigo apreciar a comida**  I am unable to enjoy food |  |  |  |  |  |
| **(12) Minha saliva torna-se espessa/pegajosa e eu preciso cuspir com frequência**  My saliva becomes thick/sticky and need to spit out frequently |  |  |  |  |  |
| **(13) Tenho mudanças de paladar**  I have taste changes |  |  |  |  |  |
| **(17) Eu modifico minha dieta (Ex.: tipo de comida, textura e tamanho)**  I modify my diet (e.g. food type, texture and size) |  |  |  |  |  |
| 3. **FUNÇÃO SOCIAL**   **Social function** | | | | | |
| **(20) A qualidade da minha voz piorou**  I speak with lower quality/voice |  |  |  |  |  |
| **(21) Tenho dificuldades para falar**  I have difficulty in talking |  |  |  |  |  |
| **(22) Preciso usar outros meios para me comunicar com outras pessoas**  **(Ex.: papel/caneta, linguagem corporal)**  I need to use other means (e.g.paper/pen, body language) to communicate with others |  |  |  |  |  |
| **(24) A dificuldade em falar me deixa angustiado(a)**  Speacking difficulty makes me distressed |  |  |  |  |  |

| **DIMENSÕES**  **Dimensions** | **CLASSIFICAÇÃO****  **Scores** | | | | | | | **PONTUAÇÃO**  **Total score** |
| --- | --- | --- | --- | --- | --- | --- | --- | --- |
|  | **1** | | **2** | **3** | **4** | | |  |
| 4. **DEGLUTIÇÃO**   **Swallowing** | | | | | | | | |
| **(27) Tenho desconforto na garganta**  I have throat discomfort |  |  | |  | |  |  | |
| **(28) Tenho dificuldade para engolir líquidos (Ex.: água, suco, sopa)**  I have difficulty in swallowing liquids (e.g. water, juice, soup) |  |  | |  | |  |  |  |
| **(30) Engasgo facilmente ao engolir**  I feel easily choked while swallowing |  |  | |  | |  |  |  |
| **(31) Tenho dificuldade para engolir a saliva**  I have difficulty in swallowing saliva |  |  | |  | |  |  |  |

**** 01:** De modo algum **(not at all)**; **02:** Um pouco **(a little bit)**; **03:** Razoável **(quite a bit)**; **04:** Muito **(very much)**

**Note:** This file presents the Brazilian shortened version of the OMQoL questionnaire, displaying both the items in Portuguese and their English version to facilitate evaluation and comprehension by international readers.

**Supplementary File 2. Original Oral Mucositis Quality of Life (OMQoL) questionnaire (Cheng et al.).**

| **Dimensions** | **Scores**** | | | | ******* |
| --- | --- | --- | --- | --- | --- |
|  | **1** | **2** | **3** | **4** |  |
| **1. SYMPTOMS** | | | | | |
| 1. I have swelling inside my mouth |  |  |  |  |  |
| 2. I have mouth ulcer |  |  |  |  |  |
| 3. Mouth pain makes me distressed |  |  |  |  |  |
| 4. I have oozing/bleeding on my lips, or inside my mouth |  |  |  |  |  |
| 5. I feel discomfort while tooth brushing/mouth rinsing |  |  |  |  |  |
| 6. Mouth pain makes me have trouble sleep |  |  |  |  |  |
| 7. I have mouth pain |  |  |  |  |  |
| 8. I have burning sensation inside my mouth |  |  |  |  |  |
| 9. I have difficulty in opening my mouth |  |  |  |  |  |
| **2. DIET** | | | | | |
| 10. I am unable to enjoy food |  |  |  |  |  |
| 11. I reduce outside social dining due to mucosal discomfort |  |  |  |  |  |
| 12. My saliva becomes thick/sticky and need to spit out frequently |  |  |  |  |  |
| 13. I have taste changes |  |  |  |  |  |
| 14. Eating difficulty makes me distressed |  |  |  |  |  |
| 15. I use longer time to drink/eat |  |  |  |  |  |
| 16. I have weight loss |  |  |  |  |  |
| 17. I modify my diet (e.g. food type, texture and size) |  |  |  |  |  |
| 18. I reduce my soft/solid food intake |  |  |  |  |  |
| 19. I worry my inadequate nutritional intake |  |  |  |  |  |
| **3. SOCIAL FUNCTION** | | | | | |
| 20. I speak with lower quality/voice |  |  |  |  |  |
| 21. I have difficulty in talking |  |  |  |  |  |
| 22. I need to use other means (e.g.paper/pen, body language) to communicate with others |  |  |  |  |  |
| 23. I feel embarrassed at mealtimes with my family/friends |  |  |  |  |  |
| 24. Speacking difficulty makes me distressed |  |  |  |  |  |
| 25. I do not want to talk to others (including talking on phone) due to mouth discomfort |  |  |  |  |  |
| 26. I have my expression. (including smiling to others) and communication affected |  |  |  |  |  |
| **4. SWALLOWING** | | | | | |
| 27. I have throat discomfort |  |  |  |  |  |
| 28. I have difficulty in swallowing liquids (e.g. water, juice, soup) |  |  |  |  |  |
| 29. I have difficulty in swallowing soft/solid food |  |  |  |  |  |
| 30. I feel easily choked while swallowing |  |  |  |  |  |
| 31. I have difficulty in swallowing saliva |  |  |  |  |  |

**Supplementary Table S1. Oral health characteristics and dental management of study participants at the first dental examination (**$\boldsymbol{n}\boldsymbol{=}\boldsymbol{56}$**).**

| **Oral health characteristic** | **n/N (%)** |
| --- | --- |
|  |  |
| **Timing of first dental examination** |  |
| Before treatment initiation | 18/56 (32.1) |
| After treatment initiation | 38/56 (67.9) |
|  |  |
| **Oral hygiene** |  |
| Good/satisfactory | 14/56 (25.0) |
| Regular | 25/56 (44.6) |
| Poor/unsatisfactory | 17/56 (30.4) |
|  |  |
| **Dental and periodontal findings** |  |
| Active dental caries | 14/56 (25.0) |
| Periodontal disease | 10/56 (17.9) |
| Odontogenic infectious focus | 15/56 (26.8) |
|  |  |
| **Other oral conditions** |  |
| Dental prosthesis use | 12/56 (21.4) |
| Mucosal/prosthetic trauma* | 3/32 (9.4) |
| Other oral lesion at first examination | 15/56 (26.8) |
|  |  |
| **Dental management** |  |
| Oral adequacy/dental treatment indicated | 21/56 (37.5) |
| Oral adequacy completed among those for whom it was indicated† | 0/21 (0.0) |
| Oral adequacy not completed among those for whom it was indicated† | 21/21 (100.0) |

**†** Oral adequacy (dental treatment/elimination of infection foci) was considered indicated when active dental caries, periodontal disease, or odontogenic infectious foci were diagnosed at the first dental examination. None of these participants completed the required dental procedures prior to or during the antineoplastic therapy due to late dental referral or systemic unfeasibility.

**Supplementary Table S2. Clinical characteristics and symptoms of oral mucositis at the retained WHO–OMQoL assessment (**$\boldsymbol{n}\boldsymbol{=}\boldsymbol{56}$**).**

| **Clinical characteristic** | **n/N (%) or median (IQR)** |
| --- | --- |
|  |  |
| **Lesion-oriented clinical findings** |  |
| Number of OM lesions, median (IQR)* | 2 (1–4) |
| Observed range of OM lesions | 0–10 |
| Erythema | 41/56 (73.2) |
| Oral/oropharyngeal candidiasis | 12/56 (21.4) |
| Other oral/oropharyngeal infection | 8/56 (14.3) |
|  |  |
| **Symptoms and functional findings** |  |
| Odynophagia | 30/56 (53.6) |
| Xerostomia | 1/56 (1.8) |
| Oropharyngeal involvement† | 28/56 (50.0) |
| Feeding-tube use | 7/56 (12.5) |

**Supplementary Table S3. Association between sociodemographic and clinical variables and OMQoL domain and total scores after excluding participants with non-malignant conditions (**$\boldsymbol{n}\boldsymbol{=}\boldsymbol{51}$**), Hospital das Clínicas of the UFMG, Belo Horizonte/MG, March/2024 to April/2025**

|  | Symptoms  Mean (SD)  Median (Min-Max) | *p* | Diet  Mean (SD)  Median (Min-Max) | *p* | Social Functioning Mean (SD)  Median (Min-Max) | *p* | Swallowing  Mean (SD)  Median (Min-Max) | *p* | Total Score  Mean (SD)  Median (Min-Max) | *p* |
| --- | --- | --- | --- | --- | --- | --- | --- | --- | --- | --- |
| Sex  Female      Male | 12,62 (4,96)  13,0 (5-20)  10,57 (5,14)  10,0 (5-20) | 0,211 | 13,07 (2,98)  14,0 (7-16)  11,04 (3,26)  11,0 (4-16) | 0,021 | 8,41 (4,36)  7,0 (4-16)  8,09 (4,12)  6,0 (4-15) | 0,728 | 9,21 (3,86)  9,0 (4-16)  8,61 (3,89)  8,0 (4-15) | 0,616 | 43,31 (14,14)  45,0 (20-65)  38,30 (13,92)  35,0 (19-63) | 0,210 |
| Age  < 40  40 a 59  ≥ 60 | 10,94 (5,28)  13,0 (5-18)  12,55 (5,11)  13,0 (5-20)  11,29 (5,04)  11,0 (5-19) | 0,597 | 11,81 (3,37)  11,0 (6-16)  12,32 (3,52)  13,5 (4-16)  12,36 (2,81)  13,0 (7-16) | 0,900 | 8,75 (4,38)  9,0 (4-16)  7,82 (4,19)  6,0 (4-16)  8,43 (4,30)  8,5 (4-16) | 0,905 | 9,25 (4,13)  9,5 (4-15)  9,00 (4,22)  8,5 (4-16)  8,50 (3,05)  7,0 (5-15) | 0,936 | 40,75 (15,68)  40,0 (19-62)  41,68 (14,09)  42,0 (20-65)  40,57 (13,34)  40,5 (23-63) | 0,974 |
| Diagnosis  Hematologic    Non-hematologic | 11,76 (5,27)  13,0 (5-20)  11,55 (4,63)  13,0 (5-19) | 0,969 | 12,00 (3,27)  13,0 (4-16)  12,82 (3,21)  13,0 (7-16) | 0,431 | 7,88 (4,22)  6,0 (4-16)  9,73 (4,07)  10,0 (4-16) | 0,195 | 8,85 (3,88)  9,0 (4-16)  9,27 (3,90)  8,0 (4-15) | 0,644 | 40,49 (14,56)  40,0 (19-65)  43,36 (12,78)  44,0 (25-63) | 0,532 |

|  | Symptoms  Mean (SD)  Median (Min-Max) | *p* | Diet  Mean (SD)  Median (Min-Max) | *p* | Social Functioning Mean (SD)  Median (Min-Max) | *p* | Swallowing  Mean (SD)  Median (Min-Max) | *p* | Total Score  Mean (SD)  Median (Min-Max) | *p* |
| --- | --- | --- | --- | --- | --- | --- | --- | --- | --- | --- |
| Cancer Treatment    Chemotherapy  Concurrent chemoradiotherapy | 11,59 (4,95)  13,0 (5-20)  12,67 (6,56)  13,0 (5-20) | 0,578 | 12,00 (3,36)  13,0 (4-16)  13,50 (1,76)  13,0 (11-16) | 0,392 | 8,09 (4,17)  6,5 (4-16)  9,67 (4,67)  11,0 (4-15) | 0,518 | 8,70 (3,82)  8,0 (4-16)  10,83 (3,86)  11,5 (5-15) | 0,174 | 40,37 (14,14)  40,00 (19-65)  46,67 (13,93)  44,5 (32-63) | 0,307 |
| Oral Mucositis  Grade 0      Grade 1    Grade 2    Grade 3    Grade 4 | 5,82 (1,32)  5,0 (5-9)^a^  9,20 (5,84)  5,0 (5-17)^ab^  15,50 (3,34)  16,0 (9-20)^b^  13,38 (3,87)  13,0 (5-20)^b^  12,60 (6,14)  16,0 (6-19)^ab^ | 0,001 | 9,36 (2,54)  9,0 (6-13)^a^  11,00 (3,74)  11,0 (7-15)^ab^  12,40 (3,43)  13,0 (4-15)^ab^  13,19 (2,76)  13,0 (7-16)^b^  14,80 (1,30)  15,0 (13-16)^b^ | 0,005 | 4,45 (1,50)  4,0 (4-9)^a^  6,80 (3,83)  5,0 (4-13)^ab^  9,50 (4,57)  10,0 (4-16)^b^  9,57 (3,85)  9,0 (4-16)^b^  10,20 (5,02)  12,0 (4-16)^b^ | 0,001 | 4,73 (0,90)  4,0 (4-6)^a^  7,80 (2,95)  7,0 (4-12)^ab^  9,50 (3,62)  10,5 (4-14)^b^  10,38 (3,65)  12,0 (4-16)^b^  12,20 (2,58)  13,0 (9-15)^b^ | 0,001 | 24,36 (4,08)  24,0 (19-32)^a^  34,80 (15,22)  28,0 (22-57)^ab^  46,90 (11,29)  45,5 (25-61)^b^  46,52 (11,51)  47,0 (25-65)^b^  49,80 (13,06)  51,0 (35-63)^b^ | 0,001 |

**Note:**

**SD:** Standard Deviation;

**Min:** Minimum;

**Max:** Maximum.

Different letters (^a, ab, b^) denote statistically significant differences according to the Kruskal-Wallis test for more than two categories of the independent variable
